# Supplementary material for: Hot spot prediction in protein-protein interactions by an ensemble system
Source: BMC Syst Biol. 2018 Dec 31;12(Suppl 9):132. doi: 10.1186/s12918-018-0665-8 (PMC6311905; doi:10.1186/s12918-018-0665-8)
Supplement: Supplementary file 2 — All relevant MATLAB code. A simple Matlab implement of our predictor. (ZIP 19,742 kb) [file 12918_2018_665_MOESM2_ESM.zip › Additional file 2/Read ASA/ASA website/ASEdb/3HFMH.htm]

 NetSurfP - Protein Surface Accessibility and Secondary Structure Predictions

  

|  |  |  |
| --- | --- | --- |
|  |  | NetSurfP - Protein Surface Accessibility and Secondary Structure Predictions  Technical University of Denmark |

  


---

```
# For publication of results, please cite:
# A generic method for assignment of reliability scores applied to solvent accessibility predictions.
# Bent Petersen, Thomas Nordahl Petersen, Pernille Andersen, Morten Nielsen and Claus Lundegaard
# BMC Structural Biology 2009, 9:51 doi:10.1186/1472-6807-9-51
#
# Column 1: Class assignment - B for buried or E for Exposed - Threshold: 25% exposure, but not based on RSA
# Column 2: Amino acid
# Column 3: Sequence name
# Column 4: Amino acid number
# Column 5: Relative Surface Accessibility - RSA
# Column 6: Absolute Surface Accessibility
# Column 7: Z-fit score for RSA prediction
# Column 8: Probability for Alpha-Helix
# Column 9: Probability for Beta-strand
# Column 10: Probability for Coil
E D  3HFMH                  1    0.784 112.917   1.265   0.005   0.015   0.979
B V  3HFMH                  2    0.191  29.372  -0.430   0.003   0.718   0.279
E Q  3HFMH                  3    0.436  77.941   1.413   0.001   0.900   0.099
B L  3HFMH                  4    0.131  23.895   0.635   0.001   0.900   0.099
E Q  3HFMH                  5    0.417  74.566   0.722   0.002   0.816   0.182
B E  3HFMH                  6    0.237  41.404   0.618   0.004   0.514   0.481
E S  3HFMH                  7    0.435  51.017  -0.513   0.004   0.197   0.799
E G  3HFMH                  8    0.486  38.240  -1.785   0.018   0.088   0.893
E P  3HFMH                  9    0.509  72.199  -2.310   0.018   0.047   0.935
E S  3HFMH                 10    0.498  58.307  -1.841   0.020   0.205   0.775
E L  3HFMH                 11    0.392  71.830  -0.855   0.021   0.451   0.528
E V  3HFMH                 12    0.290  44.558   0.554   0.021   0.451   0.528
E K  3HFMH                 13    0.399  82.033   1.195   0.020   0.205   0.775
E P  3HFMH                 14    0.465  66.054  -0.572   0.005   0.045   0.951
E S  3HFMH                 15    0.610  71.539  -1.905   0.005   0.045   0.951
E Q  3HFMH                 16    0.448  79.995  -0.479   0.004   0.138   0.858
E T  3HFMH                 17    0.427  59.169  -0.408   0.004   0.514   0.481
B L  3HFMH                 18    0.135  24.773   0.680   0.002   0.816   0.182
E S  3HFMH                 19    0.425  49.857   0.904   0.001   0.900   0.099
B L  3HFMH                 20    0.071  13.073   0.520   0.001   0.900   0.099
B T  3HFMH                 21    0.182  25.257   0.907   0.001   0.900   0.099
B C  3HFMH                 22    0.039   5.448  -0.008   0.001   0.959   0.040
B S  3HFMH                 23    0.288  33.789   0.568   0.001   0.959   0.040
B V  3HFMH                 24    0.096  14.786  -0.321   0.001   0.900   0.099
B T  3HFMH                 25    0.162  22.497  -0.562   0.004   0.616   0.381
B G  3HFMH                 26    0.237  18.636  -1.957   0.004   0.197   0.799
B D  3HFMH                 27    0.241  34.685  -1.327   0.005   0.336   0.660
B S  3HFMH                 28    0.170  19.865  -1.450   0.004   0.514   0.481
B I  3HFMH                 29    0.106  19.628  -1.323   0.021   0.451   0.528
B T  3HFMH                 30    0.197  27.324  -1.477   0.021   0.279   0.699
B S  3HFMH                 31    0.245  28.714  -1.326   0.018   0.088   0.893
B D  3HFMH                 32    0.114  16.499  -0.271   0.019   0.141   0.840
B Y  3HFMH                 33    0.084  18.015   0.106   0.022   0.359   0.619
B W  3HFMH                 34    0.028   6.758   0.314   0.021   0.756   0.223
B S  3HFMH                 35    0.048   5.614  -0.143   0.021   0.756   0.223
B W  3HFMH                 36    0.037   8.802   0.335   0.021   0.756   0.223
B I  3HFMH                 37    0.037   6.789   0.316   0.018   0.846   0.136
B R  3HFMH                 38    0.115  26.427   0.375   0.023   0.655   0.322
B K  3HFMH                 39    0.085  17.423   0.349   0.022   0.359   0.619
E F  3HFMH                 40    0.250  50.195   0.849   0.018   0.047   0.935
E P  3HFMH                 41    0.349  49.566   0.615   0.005   0.015   0.979
E G  3HFMH                 42    0.533  41.939  -1.503   0.018   0.019   0.964
E N  3HFMH                 43    0.411  60.200   0.418   0.018   0.019   0.964
E R  3HFMH                 44    0.431  98.630  -0.502   0.018   0.047   0.935
B L  3HFMH                 45    0.162  29.626   0.854   0.005   0.262   0.733
B E  3HFMH                 46    0.100  17.557  -0.387   0.004   0.616   0.381
B Y  3HFMH                 47    0.048  10.172   0.068   0.001   0.900   0.099
B M  3HFMH                 48    0.022   4.322   1.010   0.001   0.959   0.040
B G  3HFMH                 49    0.018   1.401   0.026   0.001   0.959   0.040
B Y  3HFMH                 50    0.085  18.100  -0.180   0.001   0.959   0.040
B V  3HFMH                 51    0.030   4.550   0.366   0.001   0.959   0.040
B S  3HFMH                 52    0.176  20.627   0.607   0.003   0.718   0.279
B Y  3HFMH                 53    0.348  74.303  -0.889   0.004   0.197   0.799
E S  3HFMH                 54    0.573  67.132  -2.321   0.005   0.015   0.979
E G  3HFMH                 55    0.481  37.847  -2.188   0.016   0.005   0.979
E S  3HFMH                 56    0.504  59.045  -0.824   0.018   0.047   0.935
E T  3HFMH                 57    0.351  48.711  -0.424   0.019   0.141   0.840
E Y  3HFMH                 58    0.446  95.289  -0.126   0.020   0.205   0.775
B Y  3HFMH                 59    0.230  49.087   0.572   0.019   0.141   0.840
B N  3HFMH                 60    0.276  40.421  -0.279   0.018   0.047   0.935
E P  3HFMH                 61    0.653  92.604  -0.757   0.018   0.047   0.935
E S  3HFMH                 62    0.417  48.837  -0.145   0.018   0.047   0.935
B L  3HFMH                 63    0.086  15.765  -0.483   0.019   0.141   0.840
E K  3HFMH                 64    0.409  84.070   0.818   0.019   0.141   0.840
B S  3HFMH                 65    0.203  23.780  -1.776   0.005   0.262   0.733
B R  3HFMH                 66    0.195  44.724   0.320   0.003   0.718   0.279
B I  3HFMH                 67    0.052   9.546  -0.544   0.001   0.900   0.099
B S  3HFMH                 68    0.117  13.712   0.126   0.001   0.900   0.099
B I  3HFMH                 69    0.049   9.102   0.598   0.001   0.900   0.099
B T  3HFMH                 70    0.093  12.927   0.305   0.003   0.718   0.279
E R  3HFMH                 71    0.270  61.853   0.775   0.022   0.552   0.426
B D  3HFMH                 72    0.251  36.169  -0.692   0.004   0.420   0.576
E T  3HFMH                 73    0.454  62.900  -0.265   0.018   0.088   0.893
B S  3HFMH                 74    0.239  28.023  -1.915   0.018   0.047   0.935
E K  3HFMH                 75    0.510 104.886   0.751   0.019   0.141   0.840
B N  3HFMH                 76    0.239  35.034  -0.357   0.005   0.336   0.660
B Q  3HFMH                 77    0.206  36.738   1.200   0.002   0.816   0.182
B Y  3HFMH                 78    0.068  14.574  -0.714   0.006   0.962   0.032
B Y  3HFMH                 79    0.167  35.773   0.556   0.001   0.959   0.040
B L  3HFMH                 80    0.043   7.892   0.795   0.001   0.959   0.040
B D  3HFMH                 81    0.176  25.333  -0.185   0.001   0.900   0.099
B L  3HFMH                 82    0.076  13.879  -0.506   0.003   0.718   0.279
E N  3HFMH                 83    0.358  52.382  -0.039   0.004   0.420   0.576
E S  3HFMH                 84    0.375  43.950  -0.820   0.004   0.197   0.799
B V  3HFMH                 85    0.113  17.368   0.049   0.019   0.141   0.840
E T  3HFMH                 86    0.481  66.729   1.017   0.018   0.088   0.893
E T  3HFMH                 87    0.360  50.001  -0.618   0.052   0.084   0.864
E E  3HFMH                 88    0.416  72.623  -0.603   0.052   0.084   0.864
B D  3HFMH                 89    0.177  25.578  -0.851   0.052   0.084   0.864
B T  3HFMH                 90    0.144  20.014   0.486   0.066   0.296   0.638
B A  3HFMH                 91    0.040   4.452  -0.313   0.021   0.756   0.223
B T  3HFMH                 92    0.084  11.637   0.739   0.018   0.846   0.136
B Y  3HFMH                 93    0.039   8.398   0.865   0.018   0.846   0.136
B Y  3HFMH                 94    0.060  12.779   0.959   0.018   0.846   0.136
B C  3HFMH                 95    0.019   2.654   1.452   0.021   0.756   0.223
B A  3HFMH                 96    0.032   3.504   0.043   0.022   0.552   0.426
B N  3HFMH                 97    0.136  19.910   0.048   0.021   0.279   0.699
B W  3HFMH                 98    0.238  57.311  -0.520   0.020   0.205   0.775
B D  3HFMH                 99    0.189  27.278  -1.276   0.018   0.088   0.893
B G  3HFMH                100    0.115   9.058  -1.117   0.019   0.141   0.840
B D  3HFMH                101    0.153  22.062  -0.959   0.021   0.279   0.699
B Y  3HFMH                102    0.114  24.405  -0.491   0.021   0.279   0.699
B W  3HFMH                103    0.256  61.568  -0.594   0.020   0.205   0.775
B G  3HFMH                104    0.267  21.005  -2.153   0.018   0.088   0.893
E Q  3HFMH                105    0.416  74.298  -1.416   0.005   0.045   0.951
B G  3HFMH                106    0.247  19.423  -0.867   0.004   0.138   0.858
B T  3HFMH                107    0.142  19.640   0.069   0.005   0.262   0.733
B L  3HFMH                108    0.248  45.317   0.072   0.004   0.514   0.481
B V  3HFMH                109    0.042   6.517  -1.531   0.004   0.616   0.381
B T  3HFMH                110    0.214  29.737  -0.465   0.021   0.756   0.223
B V  3HFMH                111    0.108  16.600  -1.233   0.021   0.756   0.223
B S  3HFMH                112    0.155  18.189  -1.180   0.021   0.451   0.528
B A  3HFMH                113    0.145  15.968  -1.377   0.019   0.141   0.840
E A  3HFMH                114    0.462  50.912  -0.414   0.018   0.088   0.893
E K  3HFMH                115    0.369  75.986  -0.669   0.018   0.047   0.935
E T  3HFMH                116    0.425  58.892  -0.958   0.018   0.088   0.893
E T  3HFMH                117    0.485  67.200  -1.721   0.020   0.205   0.775
E P  3HFMH                118    0.453  64.252  -1.011   0.021   0.279   0.699
E P  3HFMH                119    0.369  52.347  -0.796   0.021   0.279   0.699
E S  3HFMH                120    0.494  57.862  -1.142   0.021   0.279   0.699
E V  3HFMH                121    0.400  61.449  -1.364   0.064   0.216   0.721
B Y  3HFMH                122    0.320  68.405  -1.085   0.056   0.142   0.802
E P  3HFMH                123    0.411  58.378  -1.730   0.056   0.142   0.802
E L  3HFMH                124    0.340  62.272  -1.832   0.020   0.205   0.775
E A  3HFMH                125    0.368  40.554  -1.197   0.018   0.088   0.893
E P  3HFMH                126    0.302  42.911  -2.300   0.018   0.047   0.935
E G  3HFMH                127    0.477  37.556  -2.372   0.018   0.047   0.935
E S  3HFMH                128    0.429  50.337  -2.117   0.018   0.047   0.935
E A  3HFMH                129    0.394  43.386  -2.161   0.018   0.088   0.893
E A  3HFMH                130    0.394  43.452  -2.046   0.018   0.088   0.893
E Q  3HFMH                131    0.474  84.710  -1.512   0.018   0.088   0.893
E T  3HFMH                132    0.610  84.538  -1.596   0.018   0.047   0.935
E N  3HFMH                133    0.582  85.190  -2.386   0.005   0.045   0.951
E S  3HFMH                134    0.399  46.821  -1.408   0.004   0.085   0.910
E M  3HFMH                135    0.393  78.559   0.442   0.005   0.336   0.660
B V  3HFMH                136    0.067  10.359   0.715   0.002   0.816   0.182
B T  3HFMH                137    0.205  28.503   0.681   0.001   0.900   0.099
B L  3HFMH                138    0.031   5.658   0.220   0.001   0.900   0.099
B G  3HFMH                139    0.046   3.652   0.067   0.002   0.816   0.182
B C  3HFMH                140    0.026   3.721   0.233   0.003   0.718   0.279
B L  3HFMH                141    0.124  22.723   0.212   0.002   0.816   0.182
B V  3HFMH                142    0.043   6.594  -0.342   0.002   0.816   0.182
B K  3HFMH                143    0.245  50.417  -0.174   0.004   0.616   0.381
E G  3HFMH                144    0.348  27.364  -2.135   0.005   0.262   0.733
B Y  3HFMH                145    0.185  39.449  -0.711   0.005   0.262   0.733
E F  3HFMH                146    0.305  61.173  -0.536   0.004   0.197   0.799
B P  3HFMH                147    0.245  34.723  -1.671   0.004   0.197   0.799
E E  3HFMH                148    0.533  93.063  -1.048   0.004   0.138   0.858
E P  3HFMH                149    0.386  54.830  -1.554   0.004   0.197   0.799
B V  3HFMH                150    0.179  27.574  -0.517   0.004   0.616   0.381
B T  3HFMH                151    0.247  34.301  -0.540   0.001   0.900   0.099
B V  3HFMH                152    0.043   6.548  -0.145   0.001   0.900   0.099
B T  3HFMH                153    0.165  22.886  -0.146   0.011   0.918   0.071
B W  3HFMH                154    0.054  13.035   0.165   0.011   0.918   0.071
B N  3HFMH                155    0.235  34.345   0.059   0.002   0.816   0.182
E S  3HFMH                156    0.385  45.087  -0.664   0.004   0.616   0.381
E G  3HFMH                157    0.365  28.718  -0.460   0.004   0.197   0.799
E S  3HFMH                158    0.527  61.706   0.213   0.005   0.015   0.979
E L  3HFMH                159    0.488  89.280  -0.875   0.005   0.015   0.979
E S  3HFMH                160    0.629  73.695  -1.943   0.005   0.045   0.951
E S  3HFMH                161    0.453  53.127   0.308   0.004   0.138   0.858
E G  3HFMH                162    0.390  30.724  -0.480   0.005   0.336   0.660
B V  3HFMH                163    0.148  22.732  -0.258   0.004   0.616   0.381
B H  3HFMH                164    0.287  52.278   0.136   0.001   0.900   0.099
B T  3HFMH                165    0.233  32.317  -0.479   0.001   0.959   0.040
B F  3HFMH                166    0.166  33.216   1.033   0.001   0.900   0.099
B P  3HFMH                167    0.156  22.108  -0.656   0.003   0.718   0.279
B A  3HFMH                168    0.255  28.068  -0.830   0.002   0.816   0.182
B V  3HFMH                169    0.151  23.178  -0.790   0.002   0.816   0.182
E L  3HFMH                170    0.350  64.103  -0.254   0.004   0.616   0.381
E Q  3HFMH                171    0.604 107.928  -1.614   0.005   0.262   0.733
E S  3HFMH                172    0.738  86.447  -1.376   0.005   0.015   0.979
E D  3HFMH                173    0.517  74.500  -2.326   0.005   0.045   0.951
E L  3HFMH                174    0.420  76.920   0.244   0.004   0.514   0.481
B Y  3HFMH                175    0.215  45.881  -0.552   0.002   0.816   0.182
B T  3HFMH                176    0.179  24.758  -0.338   0.001   0.900   0.099
B L  3HFMH                177    0.131  24.004  -1.098   0.001   0.900   0.099
B S  3HFMH                178    0.183  21.506  -0.239   0.001   0.959   0.040
B S  3HFMH                179    0.031   3.586  -0.903   0.001   0.959   0.040
B S  3HFMH                180    0.167  19.584   0.204   0.001   0.959   0.040
B V  3HFMH                181    0.037   5.702  -0.374   0.001   0.959   0.040
B T  3HFMH                182    0.190  26.353   0.988   0.001   0.959   0.040
B V  3HFMH                183    0.046   7.039   0.758   0.001   0.900   0.099
E P  3HFMH                184    0.297  42.158   1.250   0.004   0.616   0.381
E S  3HFMH                185    0.349  40.868  -0.109   0.020   0.205   0.775
E S  3HFMH                186    0.693  81.255  -0.386   0.004   0.197   0.799
E P  3HFMH                187    0.581  82.472   0.095   0.004   0.138   0.858
B R  3HFMH                188    0.234  53.655  -1.202   0.004   0.085   0.910
E P  3HFMH                189    0.740 104.992   1.093   0.005   0.045   0.951
E S  3HFMH                190    0.698  81.817   0.749   0.005   0.045   0.951
E E  3HFMH                191    0.454  79.296   0.713   0.004   0.420   0.576
E T  3HFMH                192    0.397  55.064   1.428   0.001   0.900   0.099
B V  3HFMH                193    0.069  10.682   0.799   0.001   0.959   0.040
E T  3HFMH                194    0.305  42.317   1.693   0.001   0.959   0.040
B C  3HFMH                195    0.023   3.201   0.651   0.001   0.959   0.040
E N  3HFMH                196    0.351  51.328   1.131   0.001   0.959   0.040
B V  3HFMH                197    0.070  10.774  -0.603   0.001   0.900   0.099
E A  3HFMH                198    0.504  55.541   0.579   0.001   0.900   0.099
B H  3HFMH                199    0.185  33.688   0.163   0.004   0.616   0.381
E P  3HFMH                200    0.663  94.080   0.357   0.005   0.045   0.951
E A  3HFMH                201    0.718  79.146  -1.506   0.005   0.015   0.979
E S  3HFMH                202    0.553  64.823  -0.687   0.004   0.085   0.910
E S  3HFMH                203    0.571  66.968  -0.201   0.004   0.138   0.858
E T  3HFMH                204    0.435  60.307   0.469   0.005   0.336   0.660
E K  3HFMH                205    0.581 119.491   1.570   0.003   0.718   0.279
B V  3HFMH                206    0.120  18.490  -0.330   0.001   0.900   0.099
E D  3HFMH                207    0.648  93.420   0.160   0.001   0.900   0.099
E K  3HFMH                208    0.288  59.344   1.228   0.002   0.816   0.182
E K  3HFMH                209    0.599 123.276   0.921   0.004   0.616   0.381
E I  3HFMH                210    0.267  49.432   0.428   0.005   0.336   0.660
E V  3HFMH                211    0.481  73.914   0.330   0.004   0.138   0.858
E P  3HFMH                212    0.528  74.866   0.157   0.004   0.085   0.910
E R  3HFMH                213    0.668 152.903  -0.240   0.018   0.047   0.935
E D  3HFMH                214    0.642  92.584   0.120   0.018   0.047   0.935
E C  3HFMH                215    0.538  75.535  -1.824   0.000   0.000   0.999

Explain the output.  Go back.
```

---
